# Microbial activity contributes to spatial heterogeneity of wetland methane fluxes

**DOI:** 10.1128/spectrum.02714-23

**Published:** 2023-09-20

**Authors:** Wyatt Arnold, Meghan Taylor, Mark Bradford, Peter Raymond, Jordan Peccia

**Affiliations:** 1 Department of Chemical and Environmental Engineering, School of Engineering and Applied Science, Yale University, New Haven, Connecticut, USA; 2 Yale School of the Environment, Yale University, New Haven, Connecticut, USA; Connecticut Agricultural Experiment Station, New Haven, Connecticut, USA

**Keywords:** 16S rRNA, archaea, bacteria, refusal depth, peat, gene expression, gene abundance, methane flux, methanogenesis, methanotrophy

## Abstract

The emission of methane from wetlands is spatially heterogeneous, as concurrently measured surface fluxes can vary by orders of magnitude within the span of a few meters. Despite extensive study and the climatic significance of these emissions, it remains unclear what drives large, within-site variations. While geophysical factors (e.g., soil temperature) are known to correlate with methane (CH_4_) flux, measurable variance in these parameters often declines as spatial and temporal scales become finer. As methane emitted from wetlands is the direct, net product of microbial metabolisms which both produce and degrade CH_4_, it stands to reason that characterizing the spatial variability of microbial communities within a wetland—both horizontally and vertically—may help explain observed variances in flux. To that end, we surveyed microbial communities to a depth of 1 m across an ombrotrophic peat bog in Maine, USA using amplicon sequencing and gene expression techniques. Surface methane fluxes and geophysical factors were concurrently measured. Across the first meter of peat at the site, we observed significant changes in the abundance and composition of methanogenic taxa at every depth sampled, with variance in methanogen abundance explaining 70% of flux heterogeneity at a subset of plots. Among measured environmental factors, only peat depth emerged as correlated with flux, and had significant impact on the abundance and composition of methane-cycling communities. These conclusions suggest that a heightened awareness of how microbial communities are structured and spatially distributed within wetlands could offer improved insights into predicting CH_4_ flux dynamics.

**IMPORTANCE:**

Globally, wetlands are one of the largest sources of methane (CH_4_), a greenhouse gas with a warming impact significantly greater than CO_2_. Methane produced in wetlands is the byproduct of a group of microorganisms which convert organic carbon into CH_4_. Despite our knowledge of how this process works, it is still unclear what drives dramatic, localized (<10 m) variance in emission rates from the surface of wetlands. While environmental conditions, like soil temperature or water table depth, correlate with methane flux when variance in these factors is large (e.g., spring vs fall), the explanatory power of these variables decline when spatial and temporal scales become smaller. As methane fluxes are the direct product of microbial activity, we profiled how the microbial community varied, both horizontally and vertically, across a peat bog in Maine, USA, finding that variance in microbial communities was likely contributing to much of the observed variance in flux.

## INTRODUCTION

Methane (CH_4_) emitted from wetlands and inland waters constitutes more than 30% of global emissions (1, 2). These emissions are forecast to grow as a changing climate unlocks carbon stored in subarctic permafrost, and increasing temperatures accelerate emissions from pre-existing sources (3, 4). The net amount of methane released from these systems is the direct product of diverse microbial communities which are capable of producing and degrading CH_4_ under a wide range of geophysical and geochemical conditions (5). The quantitative characterization of these communities—which underpin wetland CH_4_ flux—is necessary to better constrain global flux estimates (6). Well-recognized uncertainties on the scale of 100 Tg CH_4_ yr^−1^ still persist in current models, due in part to large variability in wetland flux rates observed across spatial and temporal scales (7, 8).

Wetland CH_4_ emissions are known to fluctuate considerably with location and season (9, 10), and within the same wetland, substantial (two to three orders of magnitude) concurrent spatial variance is common (11 – 14). Frequently, this variability is attributed to environmental factors including water table depth (WTD), soil temperature, plant cover, pH, soil composition, oxygen concentration, and the availability of labile carbon (15, 16). In locations where disparate fluxes have been observed, however, variance in environmental correlates can be minimal (17), and thus, tracking microbial processes and communities may provide important information for understanding fine spatial-scale flux variability. While surface (top 15–30 cm) microbial processes have become increasingly resolved (18 – 20), growing evidence suggests that important zones of methane cycling may occur deep within wetlands (21). Ground-penetrating radar (22, 23) and pore water-sampling (15) efforts have shown that wetland belowground topography can shape hydrology in ways which enhance downward transport of labile organic carbon into deep peat, encouraging methanogenesis meters below the surface. Moreover, studies in both natural and laboratory systems have demonstrated that methane fluxes may show compensatory responses due to shifts in the composition of methanogenic communities (24), suggesting quantitative measures of physical and chemical variables alone are not sufficient for characterizing methane production capacity. As microorganisms fundamentally play a central role in CH_4_ emissions, identifying how microbial community structure and activity might underpin localized variability in methane production may further improve our ability to understand, model, and predict wetland CH_4_ fluxes on a global scale (25).

We explored the spatial distribution of archaeal and bacterial communities at an ombrotrophic bog located in Maine, USA to understand how these communities might contribute to fine spatial-scale flux variability. Historical flux monitoring at the site allowed us to focus on persistently low- and high-fluxing regions in an effort to exaggerate underlying causal factors. Our work paired extensive, multi-depth microbial gene abundance and expression measurements with concurrent and prior CH_4_ flux observations. These quantitative data were further complemented by qualitative amplicon sequencing data at a subset of sites. Correlations between flux and geochemical conditions, including soil temperature, water table depth, dissolved organic carbon (DOC), soil minerals, and pore water methane, were also evaluated.

## RESULTS AND DISCUSSION

### The wetland microbiome shifts with depth

The composition of archaeal and bacterial communities was dynamic across the first meter of peat at Orient Bog, as 16S rRNA amplicon sequencing of samples taken from four sampling depths (0, 15, 45, and 90 cm from the surface) revealed distinct communities at each level. Using amplicon data produced from universal 16S primers, which targeted bacteria and some archaea (26), sampling depth emerged as a strong explanatory variable underpinning microbiome composition (adonis2, *P* < 0.01, *R* ^2^ = 0.169), with significant dissimilarity (Jensen-Shannon divergence) between each set of depths (*P* < 0.01). Using amplicon data from archaeal-specific 16S primers, sampling depth explained even more variance of community composition (adonis2, *P* < 0.001, *R* ^2^ = 0.25), with significant differences (Jensen-Shannon divergence) again present between successive depths (*P* < 0.01) (Table S1). An unsupervised clustering analysis (Louvain) run on the universal amplicon data revealed distinct groupings that corresponded well with sampling depth (Fig. S1).

The greatest magnitude of change in the wetland microbiome occurred over the first 45 cm, possibly due to increasing moisture and the emergence of anoxic microsites (27), both of which would have allowed for a greater diversity of metabolisms. Smaller yet still significant differences were present between regions likely to have been entirely anoxic (e.g., 45 cm vs 90 cm) (Table S1). These community differences are consistent with the location of the water table, which was at 20.0 cm below the surface during sample collection. Dissolved oxygen (DO) levels, measured at 15 cm and 45 cm, suggested predominately oxic conditions nearer the surface (15 cm), with conditions transitioning to anoxia by 45 cm, as the average pore water DO dropped from 8.00 mg/L at 15 cm to 1.67 mg/L by 45 cm. Corroborating the impact of oxic conditions at the surface, abundances of archaeal sequences at the surface (0 cm) were lower than at all other depths (Fig. S2).

Community richness measurements also varied with sampling depth, with the 15 cm depth often emerging as the site of greatest richness (Fig. S3). Archaeal richness was lowest at the 45 cm and 90 cm sampling depths, whereas universal richness was comparably low at 0, 45, and 90 cm. As with the shifts in community composition, the richness of identified taxa at 15 cm was followed by a sharp decrease at lower depths. At 15 cm, a combination of both oxic and anoxic microenvironments may have existed (28), leading to a heightened diversity of metabolisms and species.

### Significant shifts in the abundance and diversity of methane-producing taxa occur with sampling depth

**Fig 1:**
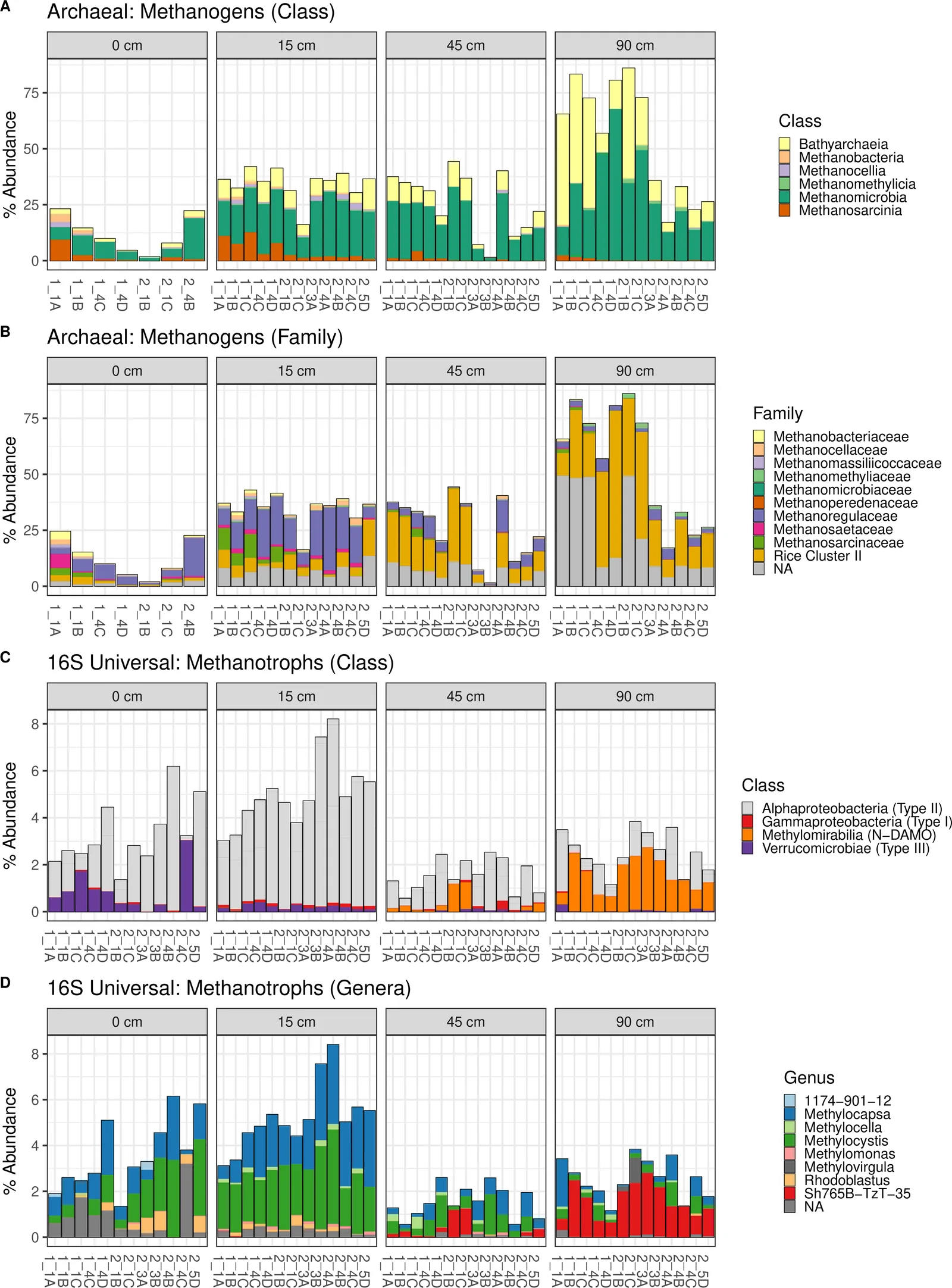
Relative abundances, based on archaeal 16S data, of known methanogenic taxa at the (**A**) class and (**B**) family levels, split by sampling depth. (**C**) Relative abundances, based on universal 16S data, of known methanotrophic classes and resolved (**D**) methanotrophic genera.

Different methanogenic taxa were found to predominate at different sampling depths, with significant shifts occurring between nearly ever depth sampled. At the near surface depth (15 cm), the dominant, resolved methanogenic family was Methanoregulaceae (Fig. 1B, 5.33%–28.25%), a member of the strictly hydrogenotrophic Methanomicrobiales order (29). For both the 0 cm and 15 cm depths, Methanoregulaceae abundance and concurrently measured mcrA DNA abundance were correlated (Table 1). As sampling depth increased, however, Rice Cluster II, another member of the Methanomicrobiales order, became dominant and accounted for, on average, 27.1% of the total archaeal community at 90 cm (Fig. 1B). The relative abundance of the Rice Cluster II family was strongly correlated with mcrA gene abundance (Table 1).

**TABLE 1:** Correlation coefficients between taxon abundances (as determined via sequencing using 16S universal or archaeal specific primers) and mcrA gene (DNA) abundance^*c*^

Primers	16S Universal				Archaeal	
Depth	0 cm	15 cm	45 cm	90 cm	15 cm	45 cm
Methanoregulaceae	0.69^*a*^	0.57^*a*^	–	–	–	–
Rice Cluster II	–	–	0.89^*b*^	0.62^*a*^	0.89^*b*^	0.78^*b*^
Bathyarchaeia	–	–	0.86^*b*^	–	–	0.91^*b*^
Thermoplasmata	–	–	–	–	–	–

^*a*^ *P*-values <0.05.

^*b*^ *P*-values <0.01.

^*c*^ *R*-values. Dash represents a lack of significant correlation (*P*-value >0.05).

Bathyarchaeia, a class which is poorly resolved at lower taxonomic levels, but whose phylum (Bathyarchaeota) is known to contain hydrogen-dependent methylotrophic methanogens (30 – 33), also became more abundant at deeper sampling depths, and, in a subset of plots at 90 cm, outcompeted Methanomicrobia, the class containing both Rice Cluster II and Methanoregulaceae (Fig. 1A). The relative abundance of the group correlated strongly with mcrA DNA (Table 1), further corroborating methanogenic activity from this clade.

Overall, the abundances of the obligately aceticlastic Methanosaetaceae family (29), and potentially aceticlastic Methanosarcinaceae family, were lower than that of Methanomicrobiales (*P* < 0.001, Welch’s *t*-test). The abundance of these aceticlastic families decreased as sample depth increased (Fig. 1B), suggesting that at our site, conditions for aceticlastic methanogenesis were more favorable near the surface. Prior work has shown that northern peatlands are dominated by non-aceticlastic pathways (34), and the minimal presence of aceticlastic groups at our site is consistent with the literature for an acidic, ombrotrophic peatland.

Archaeal taxa implicated in methane cycling at 15, 45, and 90 cm depths were all found to be less abundant at the surface (0 cm). Methanomicrobia and Bathyarchaeia surface abundances constituted just 7.1% and 1.3% of all archaeal sequences on average, respectively. Thermoplasmata, an acidophilic group, was the most abundant archaeal class across all depths, and peaked in abundance at 45 cm. While Thermoplasmata does contain the methanogenic order Methanomassiliicoccales, that group accounted for only 0%–0.7% of archaeal taxa, and broadly, Thermoplasmata did not appear to be involved in significant methane cycling. The abundance of Thermoplasmata was not correlated with mcrA abundance at any sampling depth (*P* > 0.05) (Table 1).

**Fig 2:**
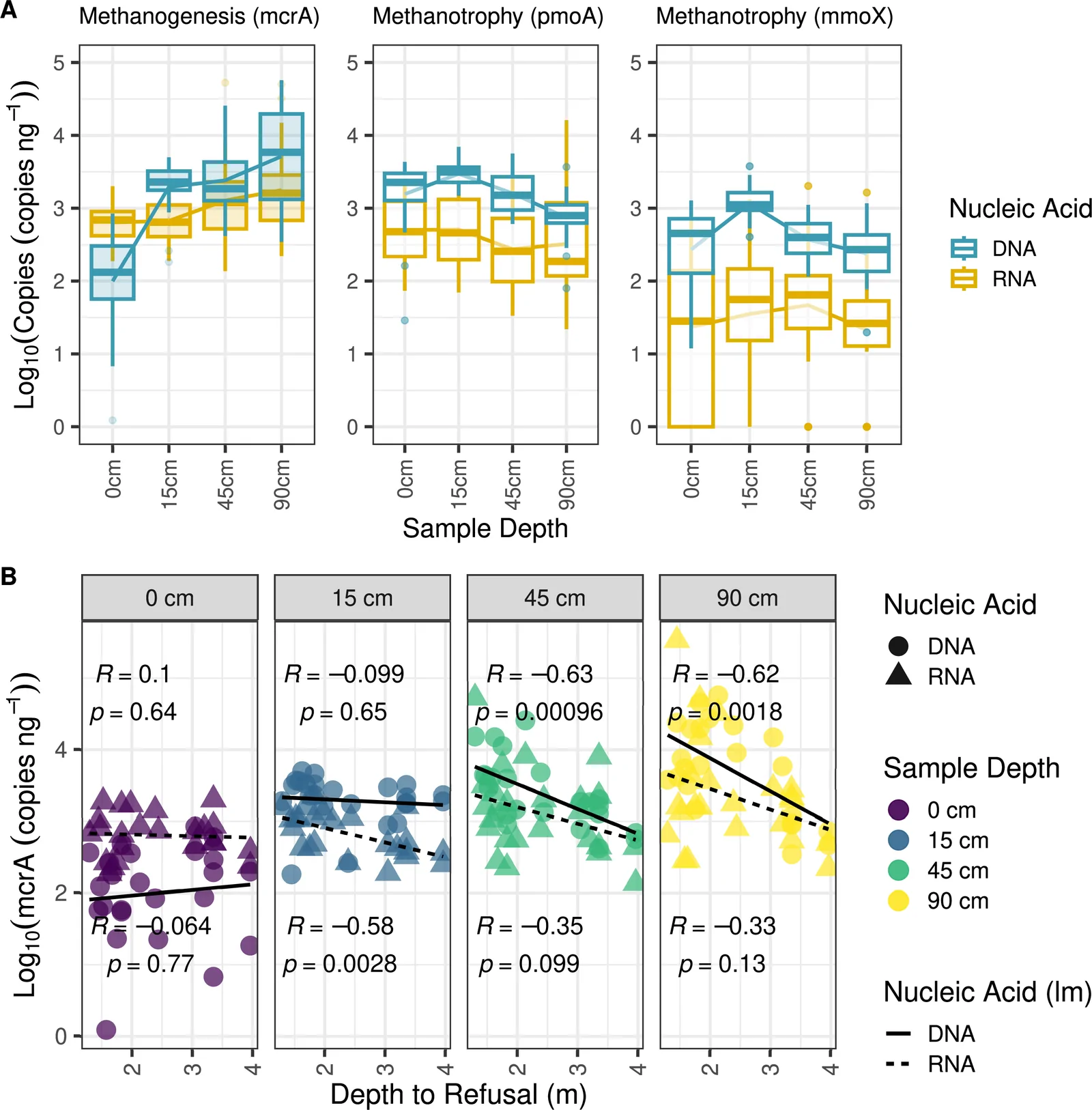
Distribution of methanogenic and methanotrophic communities across sampling depths. Both (A) the abundance of mcrA gene (DNA) and transcript (RNA) copies were observed to increase with sampling depth (solid line follows mean). For methanotrophs, both pmoA and mmoX nucleic acid abundances peaked at, or near, the 15 cm sampling depth. Relationship between (B) refusal depth (RD) and methanogen DNA and RNA, split by sampling depth. Increasing in magnitude with sample depth, a negative correlation (Pearson) emerged between methanogen abundance (DNA)/activity (RNA) and refusal depth. Correlations and significances for DNA are shown at the top of the graph, with those for RNA shown at the bottom of the graph.

As determined by droplet digital PCR (ddPCR), the absolute abundance (DNA) and activity (RNA) of methanogens generally increased with depth, peaking at 90 cm (mcrA 2.1 times higher than the next highest depth of 45 cm), our deepest sampling point (Fig. 2A). As the most abundant methanogens at these depths were in the Rice Cluster II family and the Bathyarchaeia class, it is likely that, given the observed transcriptional activity, these taxa were responsible for a large portion, if not the majority, of methane production at the site. This prominent role of Bathyarchaeia, a likely methylotrophic class, appears to support recent work on the overlooked role of methylotrophic taxa in wetlands (35).

### Significant shifts in the abundance and diversity of methane-degrading taxa also occur with sampling depth

As with the methanogens, methanotroph abundance and diversity was depth-dependent, with novel taxa still emerging in high abundances at the deepest (90 cm) sampling point. At the surface, Type III verrucomicrobial methanotrophs, primarily in the family Methylacidiphilaceae (36), competed with abundant Type II alphaproteobacterial methanotrophs for dominance, with Methylacidiphilaceae dominating in a minority of plots. By 15 cm, however, Methylacidiphilaceae abundances waned, and abundances of Methylocapsa and Methylocystis, both Type II genera, surged, peaking at 3.5% and 4.1% of total universal sequences, respectively (Fig. 1D). Across these first 15 cm, gammaproteobacterial methanotroph (Type I) abundances, mainly in the Methylomonadaceae family, were minimal but consistent. This strong selection for Type II over Type I methanotrophs is reasonable given the known qualities of ombrotrophic peat bogs (37)—methane abundance and nitrogen limitation, two conditions which both favor Type II methanotrophs (38, 39). As Type III verrucomicrobial methanotrophs can metabolize methane, hydrogen, and a variety of hydrocarbons (40, 41), their high surface abundances may imply a greater diversity of substrates were present near the surface of the bog. A majority of these methanotrophic taxa exhibited some correlation between their abundance and mmoX and/or pmoA (soluble and particulate methane monooxygenases) gene abundances (Table 2).

**TABLE 2:** Correlation coefficients between taxon abundances (from 16S universal sequencing) and functional methanotrophic gene abundances via ddPCR^*c*^

Gene	pmoA	mmoX			
Depth	90 cm	0 cm	15 cm	45 cm	90 cm
Methylocapsa	0.59^*a*^	0.60^*a*^	0.81^*b*^	–	–
Methylocystis	0.62^*a*^	0.86^*b*^	–	0.55^*a*^	–
Methylomirabilia	–	–	–	–	–
Methylacidiphilaceae	–	–	–	–	–
Methylomonadaceae	–	0.61^*a*^	–	–	–

^*a*^ *P*-values <0.05.

^*b*^ *P*-values <0.01.

^*c*^ *R*-values. Dash represents a lack of significant positive correlation (*P*-value >0.05).

At 45 cm, a marked transition occurred, as the abundance of Type II methanotrophs sharply declined, and considerable abundances of Methylomirabilia, a class known to perform nitrite-dependent anaerobic oxidation of methane (42), began to emerge (Fig. 1C). The abundance of this group continued to increase, becoming the dominant resolved methanotroph by 90 cm. The notable increase in this group at 90 cm, coupled with continued emergence of novel taxa, like Methylovirgula—a genus which may be able to oxidize both methane and sulfur (43)—suggests that important zones of methane oxidation in wetlands may extend well below its surface and into anoxic regions.

Other anaerobic methane-oxidizing taxa—especially anaerobic methane-oxidizing archaea (ANME)—were more challenging to infer given their frequent lack of taxonomic assignments or occluding clustering within methanogenic groups (44). Sulfate-reducing bacteria (SRB) are broadly known to grow in association with anaerobic methane oxidizers (45), though the true range of bacterial associates is likely diverse in wetlands (46). When considering the observed abundances of SRB (Fig. S4), ANME might only emerge in deeper (>45 cm) regions.

As quantified via ddPCR, the highest absolute abundance of methanotrophs occurred at 15 cm below the surface [1.9 times higher than the next closest depth of 45 cm based on pmoA DNA (3.1 times higher based on mmoX DNA)], with abundances generally lowest at 90 cm. Combining these observations with the ecology, we can assert that the most dominant resolved methanotrophs at Orient Bog were Type II alphaproteobacterial methanotrophs in the genera Methylocapsa and Methylocystis. As the abundance of pmoA and mmoX transcripts (Fig. 2A) also followed these same patterns of dominance, it is likely that these taxa were responsible for a significant portion of methanotrophy at the site.

Overall, the abundance, activity, and ecology of both methanotrophic and methanogenic taxa followed a less clearcut picture than the classical oxygen-focused paradigm of wetland ecology suggests. While methanotrophs were more abundant near the surface, and methanogens were more abundant at depth, neither group was ever entirely excluded from any sampling depth, supporting a more nuanced, less spatially confined hypothesis about distributions of methane-cycling taxa in wetlands (47).

### The role of refusal depth in community structure

The magnitude of change of the microbial community between sampling depths was strongly associated with the underlying refusal (peat) depth of a plot (archaea; 16S: *P* < 0.001, universal 16S: *P* < 0.01). Refusal depth varied across the site, as plots near the periphery were shallower, with refusal depths as little as 1.3 m, whereas plots near the center of the bog were considerably deeper, with refusal depths approaching 4 m. In shallow plots as compared to deep plots (refusal depth <or ≥ to the median RD), the community change was greater between sampling depths. Fig. S5 demonstrates that more variance was attributable to sampling depth for pairwise comparisons in shallow plots than for deep plots. This more dramatic rate of community change in shallow versus deep plots led to increasingly disparate communities at equivalent sampling depths between shallow and deep plots, meaning community compositions for shallow and deep plots were similar near the surface, but continually drifted apart as sampling depth increased. While similar community changes were ultimately observed across deep and shallow plots, they occurred over greater depths in deep plots.

Nutrient abundances may broadly decrease with increasing refusal depth and, in doing so, drive community variance. Studies comparing the shallow periphery of bogs to their deeper centers have found that nutrients are generally more concentrated in the shallow margins (48, 49). Prior comparisons of deep and shallow pocosins (shrub-dominated, acidic wetland) found that nutrient abundances (total soil P, K, and N:P ratios) were generally lower in deeper sites as compared to shallow sites (50, 51), and forestry literature has made special note of nutrient deficiencies (N and P) in trees growing in deep peat (52, 53). Shallow sites could see elevated nutrient levels from underlying mineral soil, or due to runoff if near the periphery of a wetland. They may also see greater nutrient loads simply because surface inputs face less dilution into the underlying peat than they would in deeper areas. Our measurements of soil composition at Orient Bog corroborated this hypothesis, as we observed either significant or trending toward significant enrichment of soil nutrients and minerals in shallow plots as compared to deep plots (Fig. 6A through C). Moreover, we conducted a tracer experiment at a subset of plots (after sampling) by applying iron (diethylenetriaminepentaacetic acid iron disodium salt [Fe-DTPA]) at the surface of the bog in order to quantify how dispersive transport of nutrients might vary between shallow and deep areas. We observed that the transport of applied iron (difference between time periods) was inversely proportional (*P* < 0.05) to refusal depth, with significant amounts of the tracer still detectable in shallow areas after a month, while the tracer signal at deep plots was small or imperceptible (Fig. 6D).

**Fig 3:**
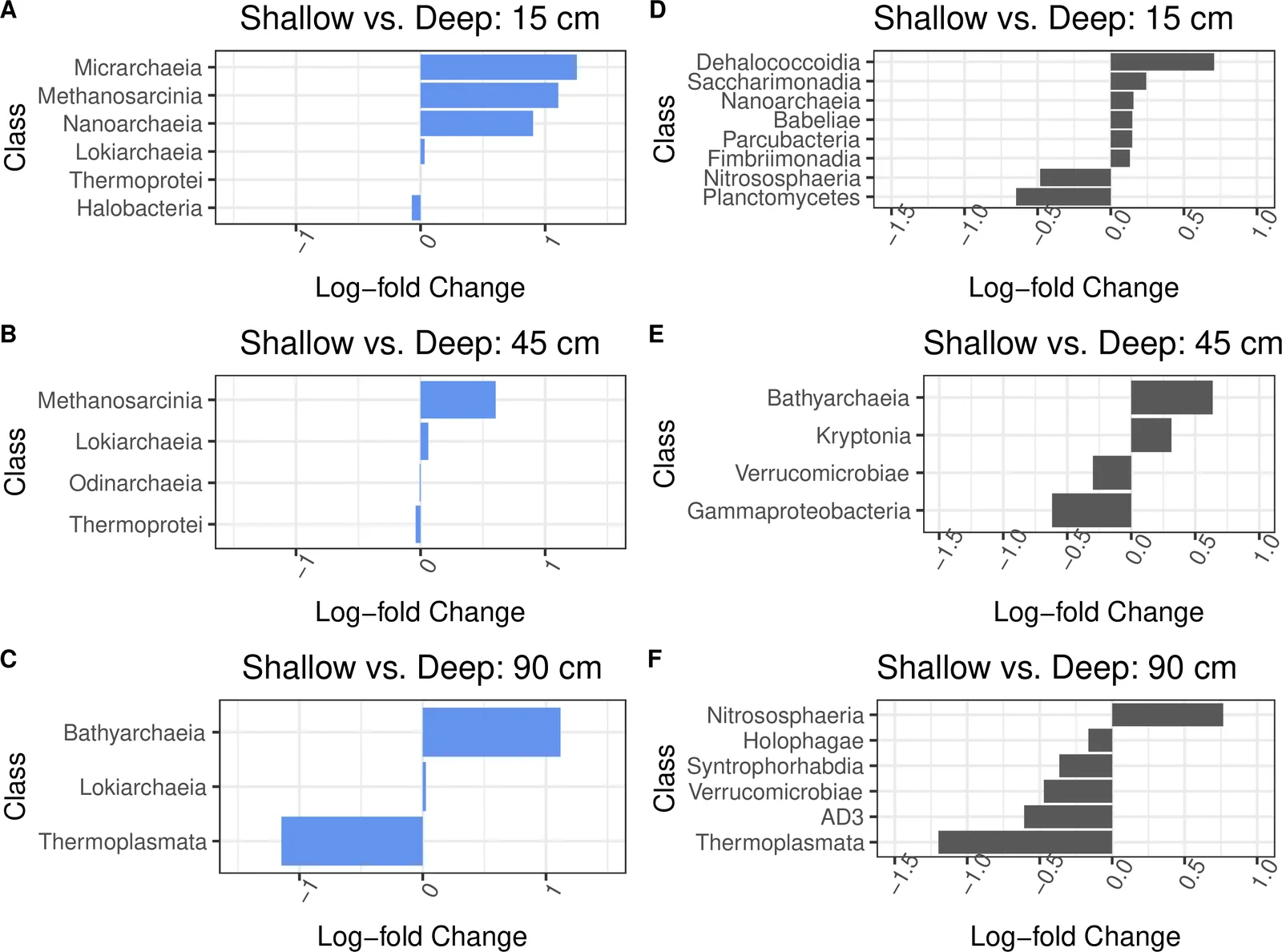
Differential abundances in taxa (at the class level, based on ANCOMBC analyses) for shallow plots relative to deep plots, split by sampling depth (refusal depth < or ≥ to the median RD). In blue, analyses (**A–C**) based on archaeal amplicon data. In gray, analyses (**D–F**) based on 16S universal amplicon data. Only taxa with significantly differential abundance (*P*-value <0.05) are displayed.

The effect of refusal depth also led to significant differences in methane-cycling taxa and activity at equivalent sampling depths between deep and shallow areas. Notably, there was a greater abundance of archaea within the order Methanosarcinales, host to aceticlastic methanogens, in shallow versus deep plots (Fig. 3A and B). This differential abundance was most evident at the 15 cm sampling depth, but persisted down to the 45 cm sampling depth. Within this order, the dominant genus was *Methanosarcina*, followed by *Methanosaeta*, an obligately aceticlastic genus. This greater abundance of aceticlastic methanogens supports our proposed characterization of shallow regions as more nutrient-rich zones, given that acetate abundance is associated with the presence of more labile organic matter (54) and high vegetative turnover (55). Additionally, significantly greater abundances of Bathyarchaeia, a dominant methanogen-containing class at our site, were observed at multiple sampling depths (45 cm and 90 cm) in shallow plots relative to deep plots (Fig. 3C and E). We observed similar trends in the quantitative abundances of mcrA genes and transcripts. Both methanogen abundance and activity at a given sampling depth decreased as refusal depth increased, with this trend most pronounced at the 45 cm and 90 cm sampling depths (Fig. 2B). In other words, the abundance and activity of methanogens measured in shallow plots were consistently higher than the abundance and activity of methanogens measured at the same depth in deep plots.

### Environmental variables—except for refusal depth—were not associated with flux heterogeneity

**Fig 4:**
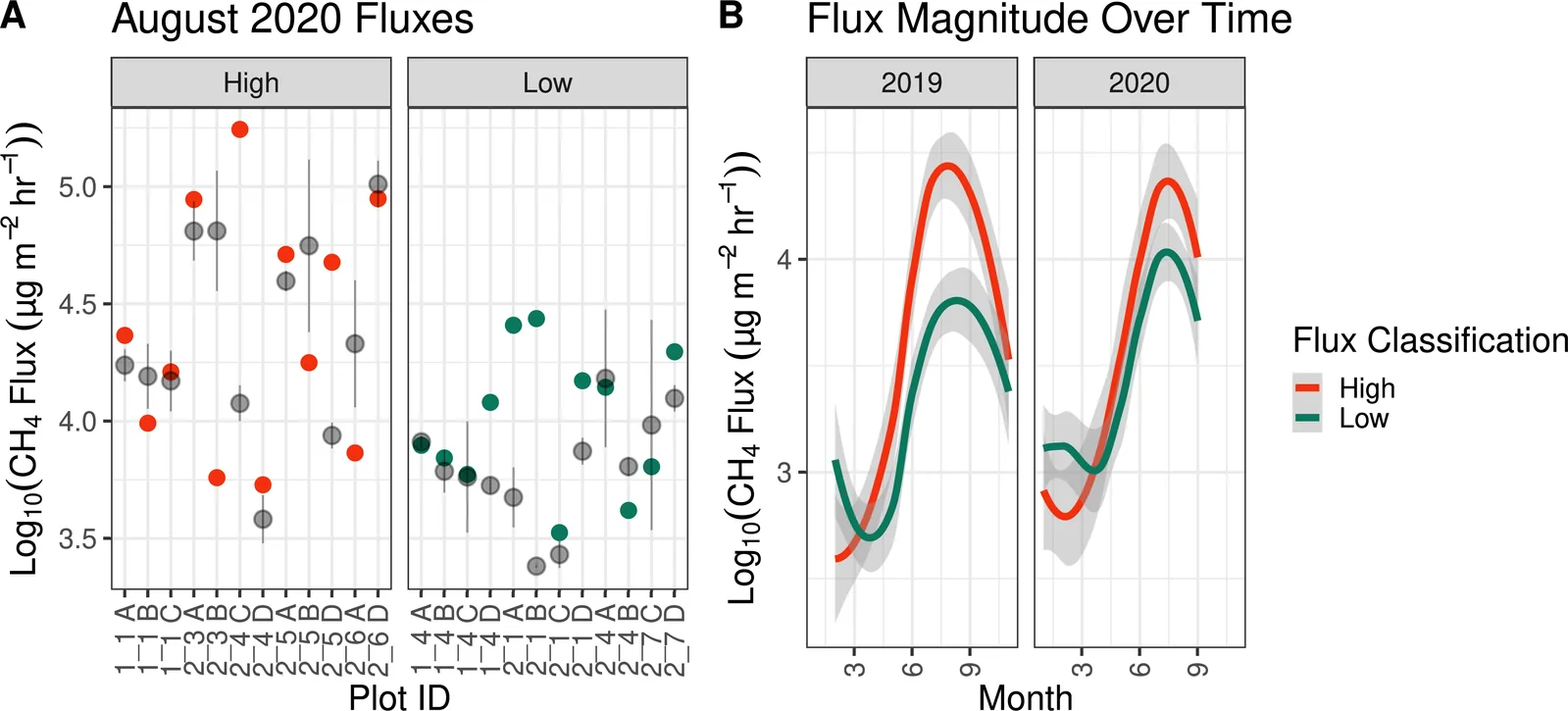
Summer methane fluxes at an ombrotrophic peat bog near Orient, Maine, USA. Prior to this 2020 study, plots were classified as either high or low fluxing depending on their mean flux relative to the overall average using data from summer (June–Sept) 2019. (**A**) Fluxes from the same 24 plots were again measured in August 2020 and were generally consistent with the magnitude of fluxes measured in August 2019 (gray). (**B**) Classifications of high-/low-fluxing plots made using summer 2019 data held for the 2020 field season (95% CI in gray), with plots classified as high fluxing consistently exhibiting higher fluxes than plots classified as low fluxing during summer months.

Methane fluxes at the site spanned nearly two orders of magnitude (min = 2,219, max = 175,202 µg CH_4_ m^−2^ h^−1^), with an among-plot SD 1.4 times greater than the mean flux of the 24 plots (Fig. 4). This range of fluxes is consistent with what has been reported for northern peatlands in the summer (11, 12, 56 – 58). The between-plot variability was not transient, with high- and low-fluxing plots persisting over time (Fig. 4B). Flux data from 2019 and 2020 showed no significant changes in mean summer flux (June–Sept) from 2019 to 2020 (*P* > 0.05) in 23 of the 24 plots. Drawing on 2019 summer flux data, plots were classified as either high (*n* = 12) or low fluxing (*n* = 12) depending on their mean flux relative to the overall average for the site.

Surface measurements of WTD and soil temperature, also taken in August, 2020, did not account for the variance in CH_4_ fluxes between plots. There was no correlation between WTD and CH_4_ flux (*R* = −0.018, *P* = 0.93) nor between soil temperature and CH_4_ flux (*R* = −0.22, *P* = 0.3), and, additionally, there was no significant difference in mean WTD or soil temperature (*P* = 0.31, *P* = 0.12) between plots classified as high fluxing or low fluxing based on prior flux data.

Pore water from the middle of the sampling window (45 cm) was also tested for dissolved organic carbon (DOC), total nitrogen (TN), pore water CH_4_, and mineral composition. Measures of DOC, TN, and pore water CH_4_ were not significantly different between high- and low-fluxing plots (*P* = 0.75, *P* = 0.23, *P* = 0.42) nor did they exhibit a correlation with methane flux (*R* = −0.19, *P* = 0.52; *R* = 0.095, *P* = 0.75; *R* = 0.1, *P* = 0.72). Additionally, there were no significant differences between high- and low-fluxing plots in terms of Cu, Fe^2+^, total iron, Mg, Ca, Mn, fluoride, chloride, or sulfate (*P* > 0.1). In a related study, plant functional composition was also found uncorrelated to flux at the site (59). While many of these factors have proven correlated with flux at larger spatial and temporal scales (7), and multi-depth measurements would be needed to fully discount their contribution here, these data suggest that they were not primary drivers of flux variability at Orient Bog.

Refusal depth, or the total depth of peat underlying a given plot, varied across Orient Bog. Plots near the periphery were shallow, with refusal depths as little as 1.3 m. Refusal depths for plots near the center of the bog were much deeper, and extended down to almost 4 m. This variability was notable, as refusal depth emerged as positively correlated with CH_4_ flux. During sampling, in August, 2020, CH_4_ fluxes were positively correlated with refusal depth (*R* = 0.28, *P* = 0.016) at Orient Bog.

### Methanogen abundance proves correlated with methane flux when accounting for refusal depth

**Fig 5:**
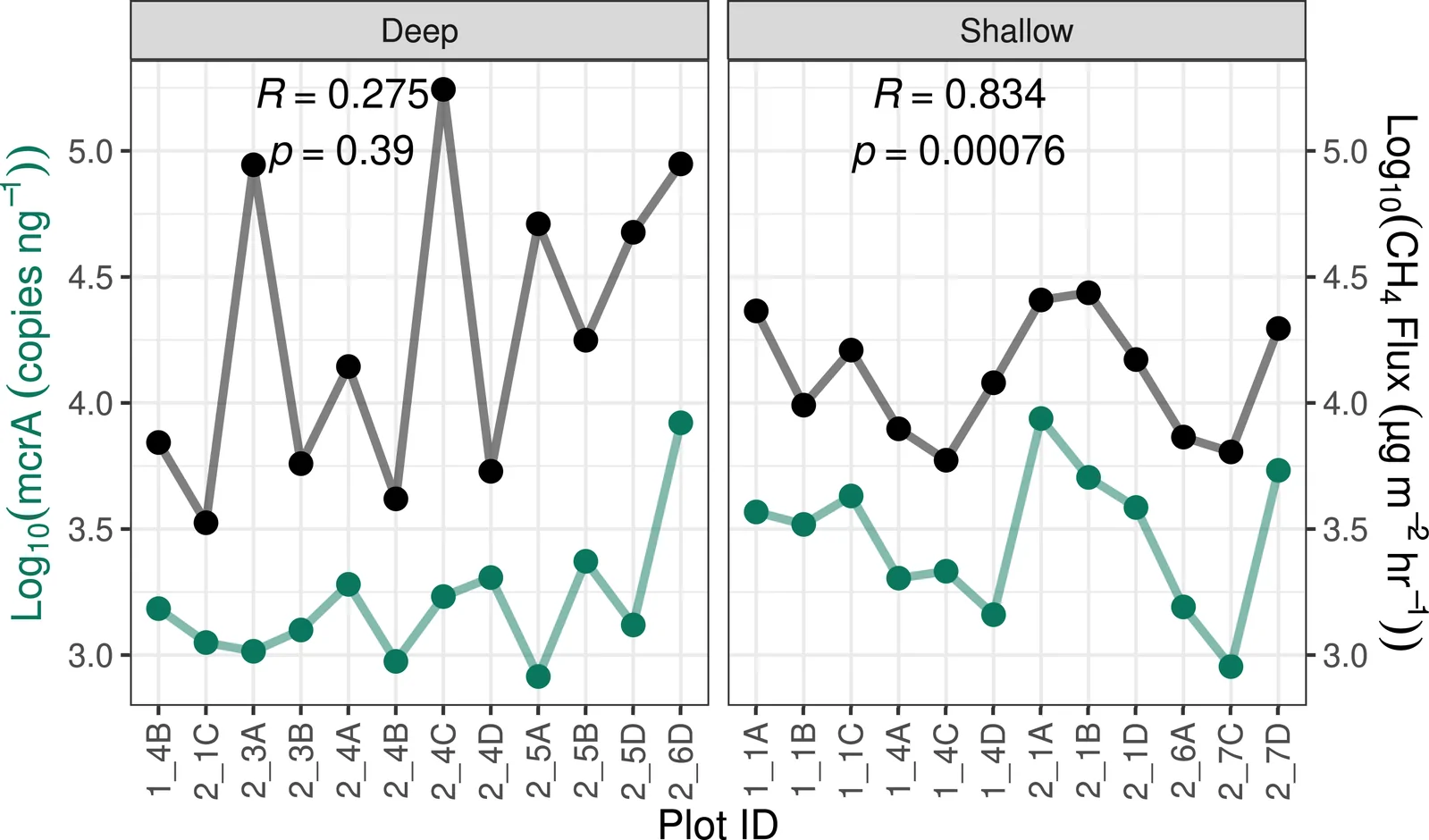
Average methanogen abundance at middle sampling depths (in green) is strongly correlated with CH_4_ flux (in black). Excluding deep plots (the microbial communities of which were likely not thoroughly surveyed by our sampling regime), there was a strong, positive correlation (Pearson) between methanogen abundances and methane flux.

Methane flux emerged as strongly linked to methanogen abundance (*R* = 0.83, *P* = 7.6e-4) in shallow plots, but not in deep plots (*P* > 0.05). Specifically, the abundance of methanogens at middle sampling depths (averaged counts from 15 cm and 45 cm) emerged as positively correlated with CH_4_ flux (Fig. 5), accounting for much of the observed spatial heterogeneity seen in shallow areas. While we hypothesize that variance in methanogen abundance also contributed to flux heterogeneity in deep plots, our sampling regime was likely insufficient (too shallow) to capture a representative picture of methanogenesis at plots with deep (>2 m) refusal depths, as a significant amount of methanogenesis may likely have been occurring in the meters beyond our sampling window.

**Fig 6:**
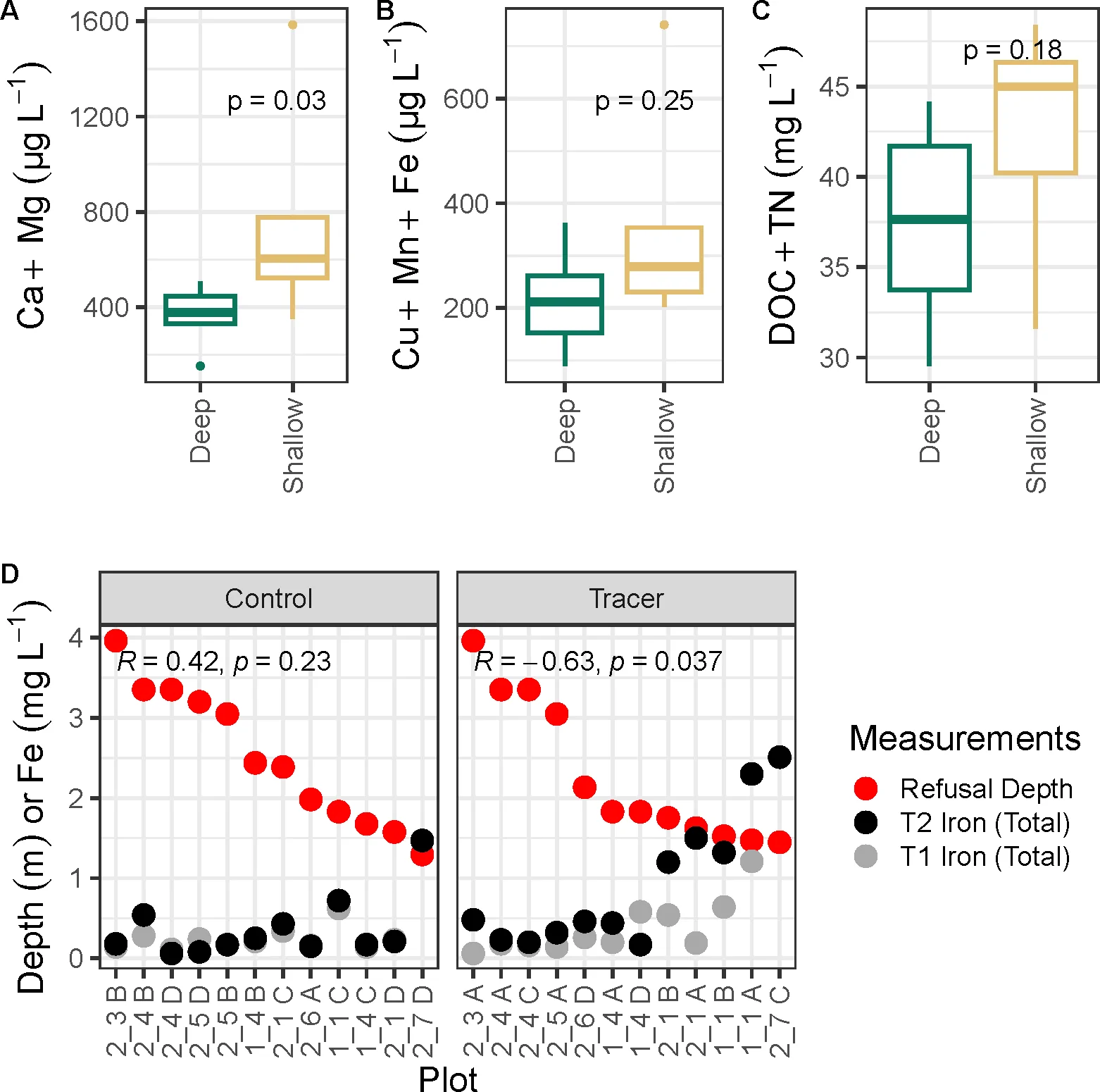
Subsurface nutrient conditions, as measured using pore water samples from 45 cm depth, split by (A) secondary nutrients (calcium and magnesium), (B) metals (copper, manganese, and iron), and (C) DOC and total nitrogen (TN). *P*-values (Wilcoxon) shown overhead for a comparison between deep and shallow plots. Results from a tracer experiment using Fe-DTPA (D) show that the transport of iron, defined as the difference in measurements between August and 1 month later in September, is inversely proportional to refusal (peat) depth.

Unexpectedly, flux and methanogen abundance at 90 cm proved to be negatively correlated (*R* = −0.85, *P* < 0.001) in shallow plots (Fig. S8). The ecology data offer some insights into the possible drivers of this behavior. Compared to deep plots at 90 cm, we saw nearly a log-fold increase in the abundances of both methylotrophic Bathyarchaeia and Nitrososphaeria—an ammonia-oxidizing class (60)—in shallow plots at 90 cm, along with a log-fold decrease in the abundance of acidophilic (61) Thermoplasmata to its lowest levels in any of the samples taken (Fig. S3 and S6). While only speculative, these taxonomic shifts imply increasing levels of nutrients, which might be the result of the underlying bedrock acting as a lower bound to catch and concentrate inputs from the surface, a process that has been observed before (22). If downward transport was rapid (Fig. 6, or surface inputs were minimal, it may limit methanogenesis nearer the surface, constraining it just to the bottom of the wetland, and resulting in the observed correlation for shallow plots.

### Community composition explains variance in diffusive methane fluxes even when accounting for known explanatory variables

**Fig 7:**
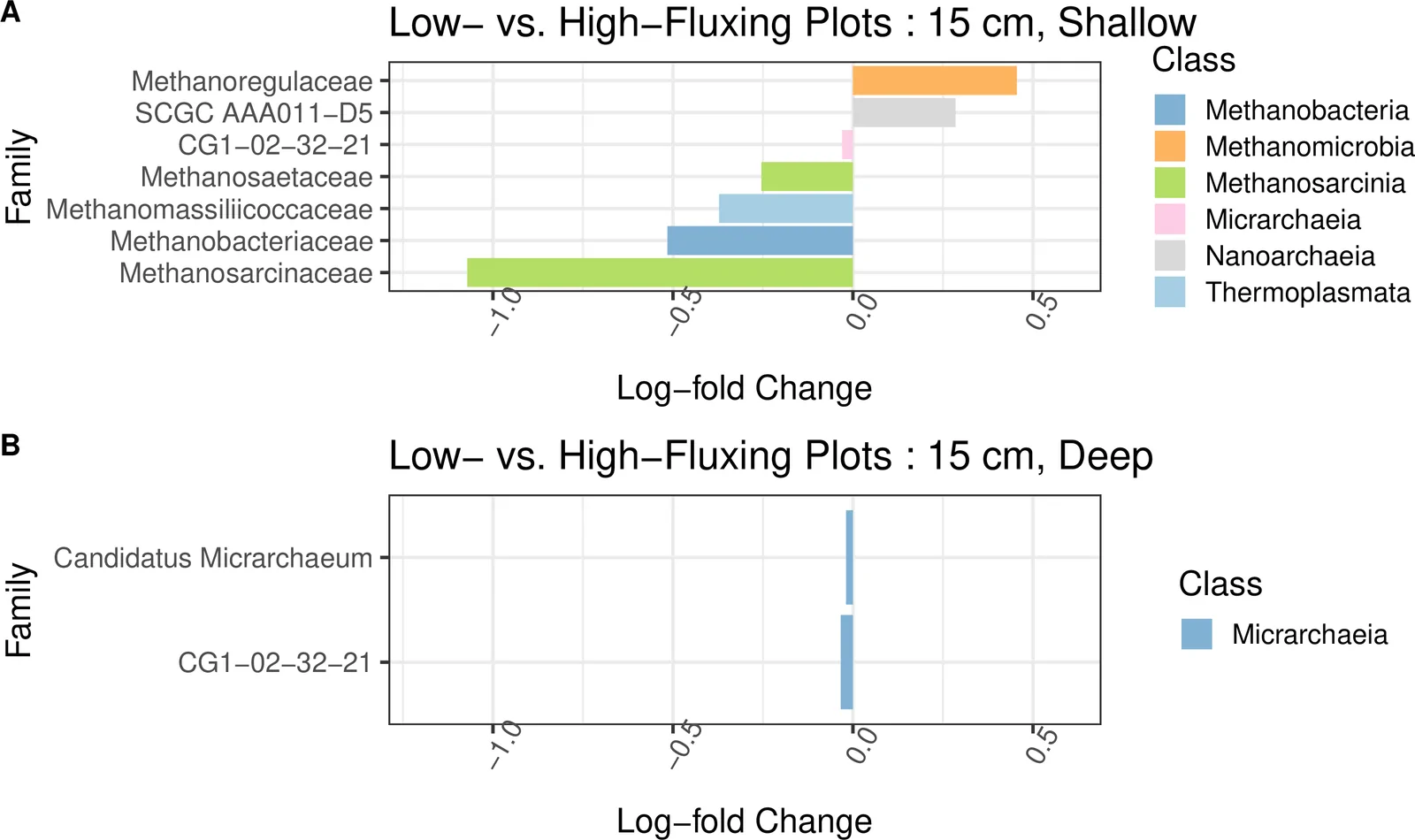
Differential abundances in taxa (based on ANCOMBC analyses) at 15 cm using archaeal-specific amplicon data, split by plots with either (A) shallow or (B) deep refusal depths. Bars display abundances of taxa in low-fluxing plots relative to their abundances in high-fluxing plots. Only taxa with significantly differential abundance (*P*-value <0.05) are displayed.

Associations between flux and community composition at each sampling depth were evaluated by determining the amount of additional variance in community structure explained by plot flux classification (“high-fluxing” vs “low-fluxing”) after accounting for variance explained by refusal depth and total methanogen abundance (mcrA DNA abundance). When evaluating the community produced using archaeal-specific primers, flux classification proved significantly associated (adonis2, sequential testing) with community composition at 15 cm (*R* ^2^ = 0.193, *P* < 0.05, Jensen-Shannon divergence). Similar trends were observed with the universal data, as flux classification was again significantly associated with community composition at both 0 cm (*R* ^2^ = 0.128, *P* < 0.001) and 15 cm (*R* ^2^ = 0.126, *P* < 0.05). At 15 cm, differential abundances of archaeal taxa were observed between high- and low-fluxing plots when accounting for refusal depth (ANCOMBC), paralleling the trend observed with mcrA abundance. In shallow plots, members of the Methanosarcinia class were found to be nearly twice as abundant in high-fluxing plots as in low-fluxing plots (Fig. 7A). Flux classification was not found to be significantly associated with community composition at either the 45 cm or 90 cm depths (*P* > 0.05).

The ability of flux classification to explain variance in community composition highlights the importance of considering the microbial community when studying wetland CH_4_ flux. While causality cannot necessarily be inferred here, the enrichment of likely aceticlastic taxa in the class Methanosarcinia in high-fluxing plots suggests that high-fluxing areas see either conditions favorable to acetogenesis or simply elevated abundances of acetate. As in this case, the abundance of certain microbial taxa likely reflects complex geophysical and geochemical conditions and, in doing so, plays a role in driving flux variations. As an extensive number of different environmental factors are hypothesized to affect methane flux (7, 15), quantifying all known correlates becomes logistically challenging, and many such variables are liable to reflect ephemeral conditions. Since community composition effectively reflects flux-relevant biogeochemical conditions, and the soil microbiome is recognized to be stable over time (62, 63)—a phenomenon confirmed at Orient Bog when repeat sampling a month later, in September (Fig. S7)—as well as robust against environmental variance (37, 64), its existing role in efforts to explain and predict wetland methane fluxes may be considerably undervalued.

### The role of methanotrophy in flux

Although methanotrophy may be able to lower CH_4_ flux, low-fluxing regions did not appear to be associated with elevated levels of methanotrophy. Rather, methanotrophy appeared constrained by methanogenesis and the supply of CH_4_. When examining correlations between methanotroph (pmoA and mmoX) and methanogen abundance (DNA) and activity (RNA) at all four sampling depths (0, 15, 45, and 90 cm), 10 out of the 16 correlations showed a significant (*P* < 0.05) positive correlation between methanotroph and methanogen abundances and activity. Moreover, none of the correlations revealed an inverse relationship between the two groups (*P* > 0.05). Neither methanotroph (pmoA and mmoX) activity nor abundance was significantly (*P* > 0.05) elevated at low-fluxing plots relative to high-fluxing plots. This is further supported by the amplicon data, as no discernable differences in methanotroph relative abundances were present between low- and high-fluxing sites at any sampling depth (*P* > 0.05).

### Conclusions

While the archaeal and bacterial taxa responsible for methanogenesis and methanotrophy within wetlands are increasingly well defined, the within-wetland spatial distribution of these microbial communities is less resolved. This work demonstrates that the methane-cycling microbiome was highly dynamic throughout the first meter of peat at our site, with different dominant methanogens and methanotrophs appearing at nearly every depth sampled. Surprisingly, depth to refusal also exerted clear control over community composition, as well as methanogen abundance, activity, and even methane flux. While many recognized environmental drivers of flux were evaluated, we demonstrated that fine spatial-scale flux variability at an ombrotrophic bog was most attributable to microbial processes. Given this relationship between microbial abundance, activity, community composition, and flux, the continued quantitative study of the wetland microbiome—beyond surficial depths—is likely to continue to improve our understanding of the composite controls over wetland CH_4_ fluxes.

## MATERIALS AND METHODS

### Site description

The raised peat bog at Orient, Maine, USA is located within the Mattawamkeag River watershed (45°51’21”N, 67°53’58”W), at 126 m elevation encompassing an area of 0.41 km^2^. This area is characterized by the US Fish and Wildlife Service National Wetlands Inventory as an acidic freshwater forested and shrub wetland with seasonally saturated substrate. The bog vegetation forms an open canopy and is dominated by stunted trees and saplings [*Picea mariana* (Mill.) Britton*,* Sterns & Poggenb. var. *mariana*], dwarf shrubs [e.g., *Chamaedaphne calyculata* (L.) Moench, Kalmia polifolia, Wangenh.], and some tussock-forming sedges [*Eriophorum vaginatum,* L. var. *spissum* (Fernald) B. Boivin]. The ground cover is dominated by *Sphagnum* mosses.

Environmental variables, microbial measurements, and CH_4_ fluxes were assessed across two transects, which were laid out in approximate parallel lines stretching from the southeastern (SE) to the northwestern (NW) bog edges, along the longer axis of the oblong bog. Transect 1 was southerly and ran across more open, shallower peat, and was approximately 700 m in length. Transect 2 ran ~760 m in length and traversed through more forested and deeper peat, crossing the highest point of the domed bog. These transects were ~200 m apart at the narrowest point in the SE, and ~280 m at the widest in the NW end. Along each transect were seven groups of plots, arranged in ~120 m intervals numbered from 1 (SE) to 7 (NW). Within each group, there were three or four plots, randomly arranged between ~1.5 m and 11 m apart. The plots themselves were demarcated using polyvinyl chloride (PVC) collars of 45 cm diameter, which were permanently installed 8 cm into the soil layer. A map of the site and plot location are available in a related manuscript (59). Plot nomenclature followed the format: transect # _ plot #, subplot, with plot numbering increasing from the SE to NW.

While there were 55 plots in total at Orient Bog, 24 were selected for the characterization described herein. Approximately half of these plots (*n* = 13), split between deep and shallow areas, were then selected for amplicon sequencing at all four depths. The collection of 24 plots chosen was curated to include the 12 highest-fluxing and 12 lowest-fluxing plots relative to the mean 2019 summertime flux for all 55 plots at the site. By selecting this bifurcated group of plots, the goal was to exaggerate the underlying causes of flux variance to improve our ability to detect the causal factors.

### Flux measurements

Surface flux measurements were made with a Los Gatos Research (LGR) Microportable Greenhouse Gas Analyzer (M-GGA-918; Los Gatos Research, San Jose, CA, USA), and boardwalks were installed adjacent to collars so that measurements could occur without disturbance. Collars were sealed with an opaque 0.11 m^3^ chamber fitted with a fan. A closed loop was created between chamber and the LGR M-GGA as air was recirculated via inlet and outlet tubing. Between each collar measurement, the chamber was removed until CH_4_ and CO_2_ concentrations returned to background atmospheric levels. Fluxes were calculated by fitting a linear slope using 5 min of measured data. Non-zero fluxes with *R* ^2^ <75% were discarded (3% of all measurements). Monthly measurements were made at each plot, a total of 14 times between June 2019 and August 2020. Using 2019 summertime (June–September) flux data from all 55 plots at the site, plots with fluxes lower than the 2019 mean summertime flux were classified as “low-fluxing,” whereas plots with fluxes higher than the 2019 mean summertime flux were classified as “high-fluxing.”

### Peat sampling

Sediment was collected from each of the 24 plots in August 2020, and again in September 2020, using a modified stainless steel grain probe, which allowed for the collection of isolated sediment samples every 15 cm to a depth of 150 cm. The probe consists of an outer shell, containing flared openings every 15 cm, and a solid, freely rotating inner rod, which possesses matching pockets every 15 cm. The probe was inserted into the ground in the closed position (inner rod rotated so pockets were shut to prevent intra-depth contamination), the inner rod was then rotated so the pockets aligned with the openings in the outer shell, and the entire probe was rotated to force sediment into the pockets. The inner rod was then rotated to the closed position to protect the samples, and the probe retracted. Sediment samples were then sub-sampled from the pockets corresponding to 0, 15, 45, and 90 cm depths, and were immediately stored in RNA/DNA shield (Zymo, Irvine, CA, USA) until their transport back to Yale University, whereafter they were stored at −80°C until processing.

### Geophysical and geochemical measurements

Soil temperatures were measured at each plot during flux measurements at 12 cm depth. Water table depth was measured during flux measurements in ground water wells.

Depth to refusal was measured by driving lengths of threaded rod into the ground at each plot, with additional lengths of rod coupled as needed until refusal depth was reached. Depth to refusal was then measured as the length of rod below the surface, defined as the top of the overlying sphagnum mat. The end of the rod was inspected for clay or gravel residues to ensure underlying wood did not confound measurements (65, 66). Refusal depths at the site ranged from 1.3 m to nearly 4 m, and samples at locations with refusal depths ≤ median RD (2.06 m) were classified as “shallow,” whereas samples at locations with refusal depths > median RD (2.06 m) were classified as “deep.”

For pore water sampling, custom-made stainless-steel piezometers capped with 40 µm sintered bronze filters were installed in each plot to a depth of 45 cm. While 24 piezometers were installed, dry conditions in the region meant the piezometers at 10 of the plots did not yield sufficient water for analysis. At a subset of plots, shorter piezometers were also installed at a depth of 15 cm.

To quantify DOC, TN, and mineral composition, pore waters were collected via syringe into acid-washed Nalgene bottles and stored in the dark, on ice for transfer to Yale University. Upon return, the samples were acidified to pH 2 and analyzed for DOC and total dissolved nitrogen on a Shimadzu total organic carbon analyzer with a TDN (total dissolved nitrogen) module (Shimadzu Scientific Instruments, Columbia, MD). Metals were quantified via ICP-MS at the Yale Analytical and Stable Isotope Center.

Pore water samples, for dissolved oxygen measurements, were taken from 15 cm (*n* = 5) and 45 cm (*n* = 21) depths using similar piezometers. Dissolved oxygen measurements were taken using a Hach DR/890 spectrophotometer and High Range Dissolved Oxygen AccuVac Ampules (Hach, Loveland, CO).

To determine aqueous pore water concentrations of CH_4_, samples were taken from piezometers at 45 cm and we performed headspace equilibration in the field following, where water samples were shaken in a vial to equilibrate dissolved gases with the ambient headspace, and then 15 mL of equilibrated headspace gas was injected into a pre-evacuated 12 mL Exetainer vial. These gases were quantified via gas chromatograph (SRI Instruments Model 8610C, Torrance, CA). Aqueous concentrations were calculated using Henry’s law and the ideal gas law to determine gas dissolved CH_4_ concentrations in the equilibrated water samples from the gas concentrations in the equilibrated headspace (67).

For the iron tracer experiment, 4.61 g of iron, in the form of Fe-DTPA, was dissolved in deionized water and applied at each of a subset of plots with a pressurized sprayer after the conclusion of the August 2020 sampling campaign. Both prior to the iron application, and one month later, in September, 2020, pore water samples were taken from 45 cm and total iron concentrations were determined using a Hach DR/890 spectrophotometer with FerroVer Iron Reagent Powder Pillows.

### DNA and RNA extraction

Nucleic acids (RNA and DNA) were simultaneously extracted from sediment samples using Takara NuceloBond Soil Mini RNA kits in concert with NuceloBond Soil Mini DNA kits (Takara Bio USA, San Jose, CA, USA). After thawing, samples were thoroughly vortexed to homogenize and ~0.75 g (wet weight) of sample was added to extraction tubes. Samples were bead-beat for 5 min using a MiniBeadbeater (Biospec, Bartlesville, OK, USA) and then the kit’s standard nucleic acid extraction protocol was followed, with the only variation being the exclusion of Buffer OPT to maximize humic acid removal. RNA and DNA concentrations were quantified using high-sensitivity kits on a Qubit fluorometer (Thermo Fisher Scientific, Waltham, MA, USA). Prior to gene abundance/expression measurements, cDNA was synthesized from RNA using GoScript cDNA synthesis kits (Promega, Madison, WI, USA) with random primers. As a template, 7 µL of RNA was used, and samples were denatured at 80°C prior to proceeding with synthesis according to protocol.

### Gene abundance and expression measurements

The separate biological processes of methanogenesis and methanotrophy were assessed by quantifying the abundances of key functional genes and their transcripts (36, 68, 69). Gene abundances (DNA) and activities (RNA transcripts) were quantified via ddPCR using a QX200 Droplet Digital PCR System (BioRad, Hercules, CA, USA). Primers targeting the genes encoding for the alpha subunit of the methyl-coenzyme m reductase enzyme (mcrA) (68) were used to indicate methanogenesis, and primers targeting the alpha subunit of the particulate methane monooxygenase (pmoA) (69), and the soluble methane monooxygenase enzyme (mmoX) (70) were used to indicate methanotrophy. While these primer sets have been extensively used and validated in the literature (69, 71 – 73), new thermocycling conditions were derived for their use in ddPCR. Annealing temperatures for primers were first calculated *in silico* via SnapGene (GSL Biotech, Chicago, IL, USA), and were refined using gradient PCR. Each 20 µL reaction consisted of: 10 µL 2X EvaGreen Supermix (BioRad), 0.36 µL each of 10 µM forward and reverse primers (to achieve a 180 nM concentration), 7.3 µL of water, and 2 µL of template. Thermocycling conditions were optimized to minimize the effects of ddPCR “rain” without sacrificing assay sensitivity. Standards, used to evaluate assay performance, consisted of gBlocks (IDT, Coralville, IA, USA) produced using portions of either the *Methanococcus voltae* (mcrA) or *Methylococcus capsulatus* (pmoA/mmoX) genomes. Standard curves for each target were evaluated over the range from 10^1^ to 10^5^ copies, with all having an *R* ^2^ >0.95. To guarantee robust quantification of target sequences, at least 15,000 droplets were generated and read per reaction. Primer sequences and thermocycling conditions are listed in Table S2. Copy numbers were normalized against sample nucleic acid concentration (RNA or DNA ng μL^−1^).

### Sequencing

Extracted DNA was sent to The University of Minnesota Genomics Center for amplicon sequencing. Universal primers (515F/806R) targeting the V4 region of the 16S rRNA gene were used, along with archaeal-specific primers (516F/915R) targeting the V4–V5 region of 16S rRNA gene. PNA (mPNA and pPNA) blockers (PNA Bio, Newbury Park, CA, USA) were added during library construction to block amplification of plastid and mitochondrial DNA. Libraries were dual-indexed, and sequenced on a 2 × 300 bp paired-end flow cell using an Illumina MiSeq platform (Illumina, San Diego, CA).

### Bioinformatics and statistical analyses

Sequence data processing and subsequent statistical analyses were performed in R v.4.0.4 (R Core Team). Pre-processing of sequence data were accomplished using the dada2 package v.1.16.0 (74), and consisted of read quality control, amplicon sequence variant (ASV) inference, paired-end merging, chimera removal, and taxonomic assignment [clustering at 99% identity using the SILVA 138.1 database (75)]. For 16S universal amplicon data, read count was rarefied to 20,000 reads, and for 16S archaeal amplicon data, read count was rarefied to 8,000 reads. The majority of subsequent downstream analyses were performed using the phyloseq package v.1.34.0 (76). Differential abundance analyses were carried out with the ANCOMBC package v.1.0.5 (77), and diffusion map dimensional reductions were accomplished using the destiny package v.3.4.0 (78). PERMANOVA and pairwise-PERMANOVA analyses were conducted with the vegan v.2.5-7 (79) and RVAideMemoire (80) v.0.9-81 packages. Correlations between ASV relative abundances and gene copy numbers (log_10_ normalized) were accomplished by fitting a linear model and calculating subsequent Pearson correlation coefficients.

## Supplementary Material

Reviewer comments

## Data Availability

Raw 16S rRNA sequencing data, along with corresponding metadata, has been deposited at the Sequence Read Archive (SRA) hosted by NIH (BioProject: PRJNA936900). All gene expression data used in this study, along with the accompanying processing scripts, are available on Dryad.

## SUPPLEMENTAL MATERIAL

Supplemental material spectrum.02714-23-s0001.pdf Eight figures, two tables.

OPEN PEER REVIEW reviewer-comments.pdf An accounting of the reviewer comments and feedback.

ASM does not own the copyrights to Supplemental Material that may be linked to, or accessed through, an article. The authors have granted ASM a non-exclusive, world-wide license to publish the Supplemental Material files. Please contact the corresponding author directly for reuse.
