# Supplementary material for: Microbial activity contributes to spatial heterogeneity of wetland methane fluxes
Source: Microbiol Spectr. 2023 Sep 20;11(5):e02714-23. doi: 10.1128/spectrum.02714-23 (PMC10580924; doi:10.1128/spectrum.02714-23)
Supplement: Supplemental material — Eight figures, two tables. [file spectrum.02714-23-s0001.pdf]

Supplementary Information

SI Table 1. Variance explained (R^2 values) by sampling depth in pairwise comparisons. All p-values <0.01, Pairwise PERMANOVA (adonis2) using Jensen-Shannon Divergence. Comparisons using 16S universal data in grey, 16S archaeal data in blue.

Depth	0 cm	15 cm	45 cm	0 cm	15 cm	45 cm
**15 cm**	0.194	-	-	0.379	-	-
**45 cm**	0.293	0.203	-	0.358	0.201	-
**90 cm**	0.291	0.287	0.15	0.414	0.370	0.156

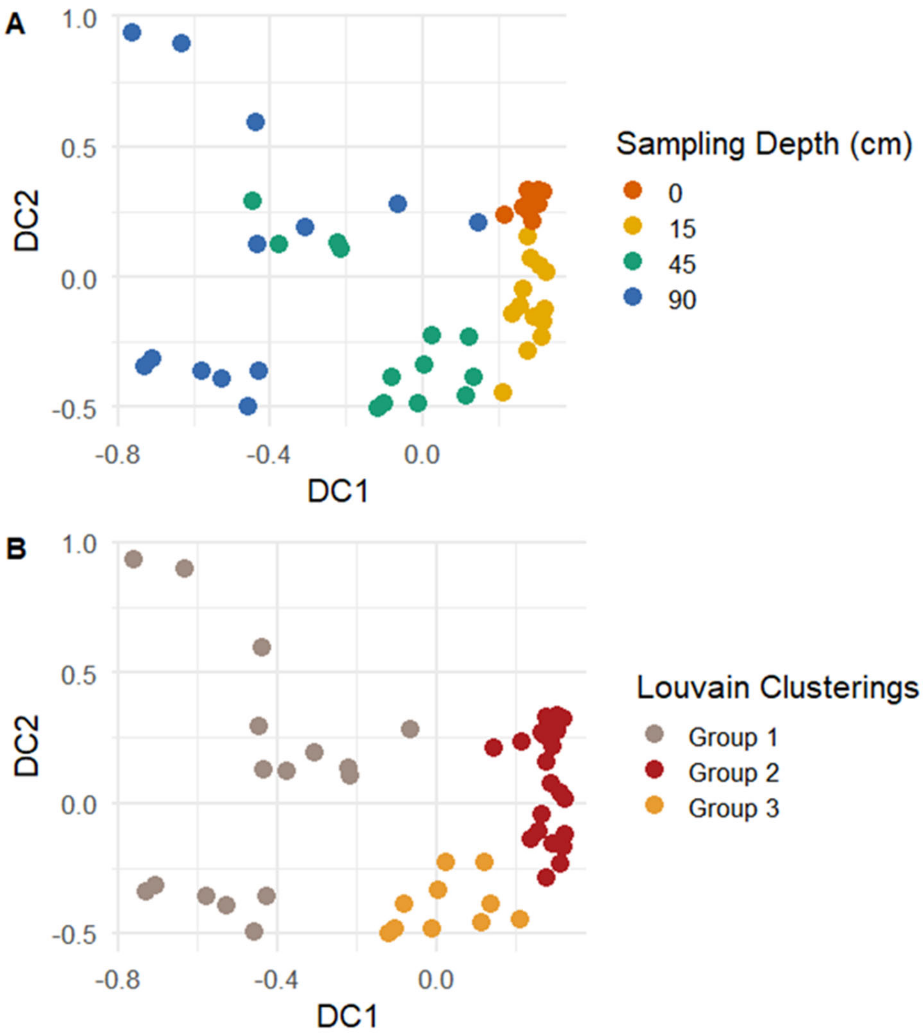

SI Figures 1a,b: Diffusion map dimensional reduction of 16S universal data (Bray-Curtis distance), first two diffusion coordinates (DC) on x- and y-axes. **a.)** Overlaying sampling depth onto the diffusion map shows that samples separate well by sampling depth. **b.)** An unsupervised clustering analysis (Louvain) run on the universal amplicon data inferred communities that matched well with sampling depth.

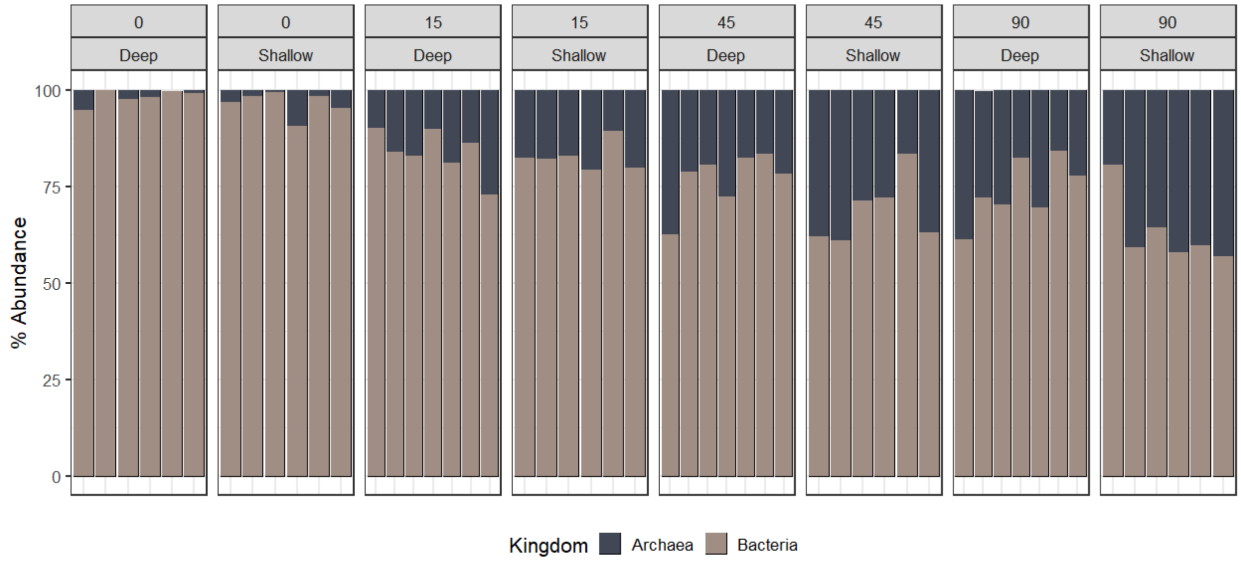

SI Figure 2: Relative abundances, at the kingdom level, produced using 16S universal amplicon data. Abundances split by sampling depth (cm) and refusal depth category.

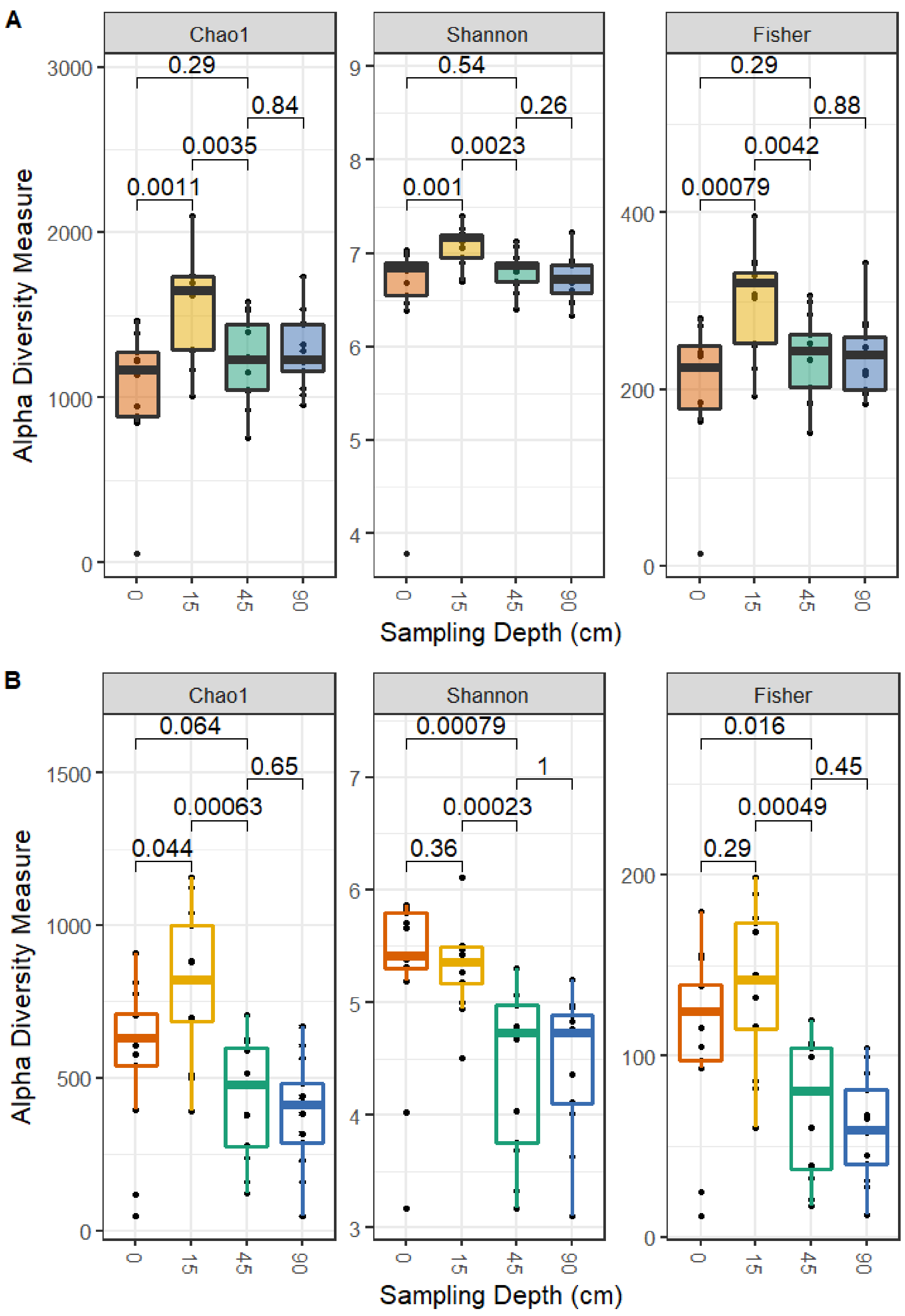

SI Fiugre 3a,b: Boxplot of three alpha-diversity metrics for **a.)** 16S universal data, and **b.)** 16S archael data, split by sampling depth (cm). Adjusted p-values for pairwise comparions listed overhead.

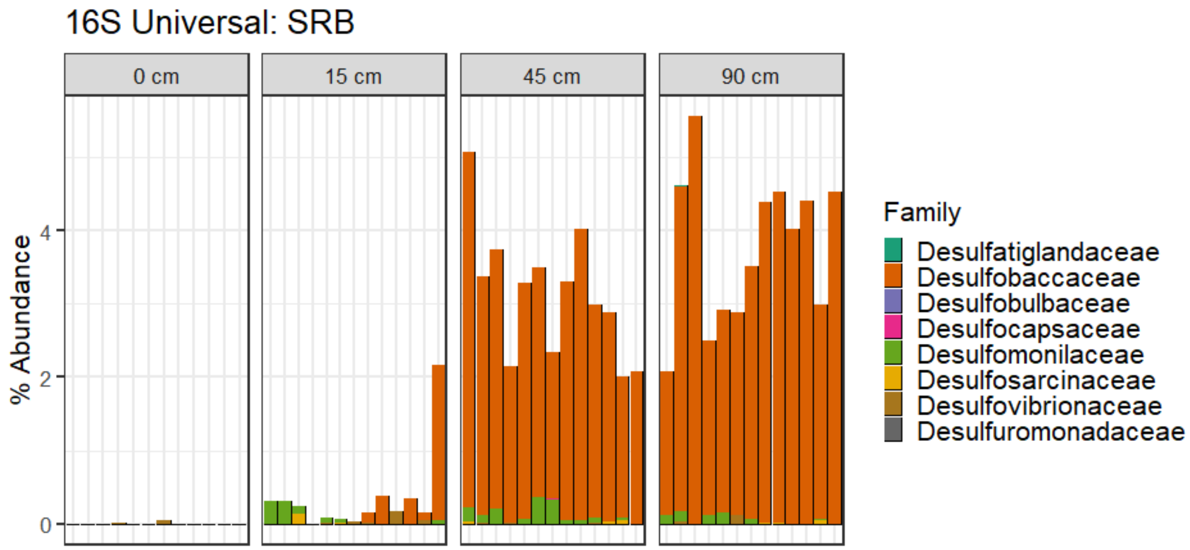

SI Figure 4: Relative abundances, based on universal 16S data, of annotated sulfur reducing bacteria, split by sampling depth.

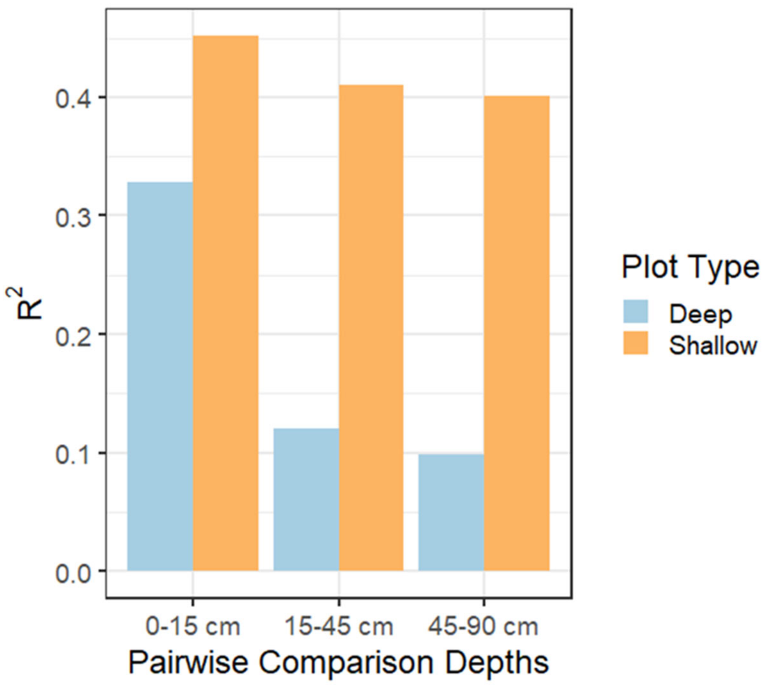

SI Figure 5: Pairwise comparisons (PERMANOVA, based on Jensen–Shannon Divergence) of community composition between adjacent sampling depths using archaeal amplicon data, with samples split into deep and shallow refusal depth. R^2 describes the amount of variance in the pairwise comparison attributable to sampling depth. Sampling depth consistently explained more of the variance for inter-depth comparisons in shallow plots vs. deep plots, indicating greater differences between sampling depths in shallow areas.

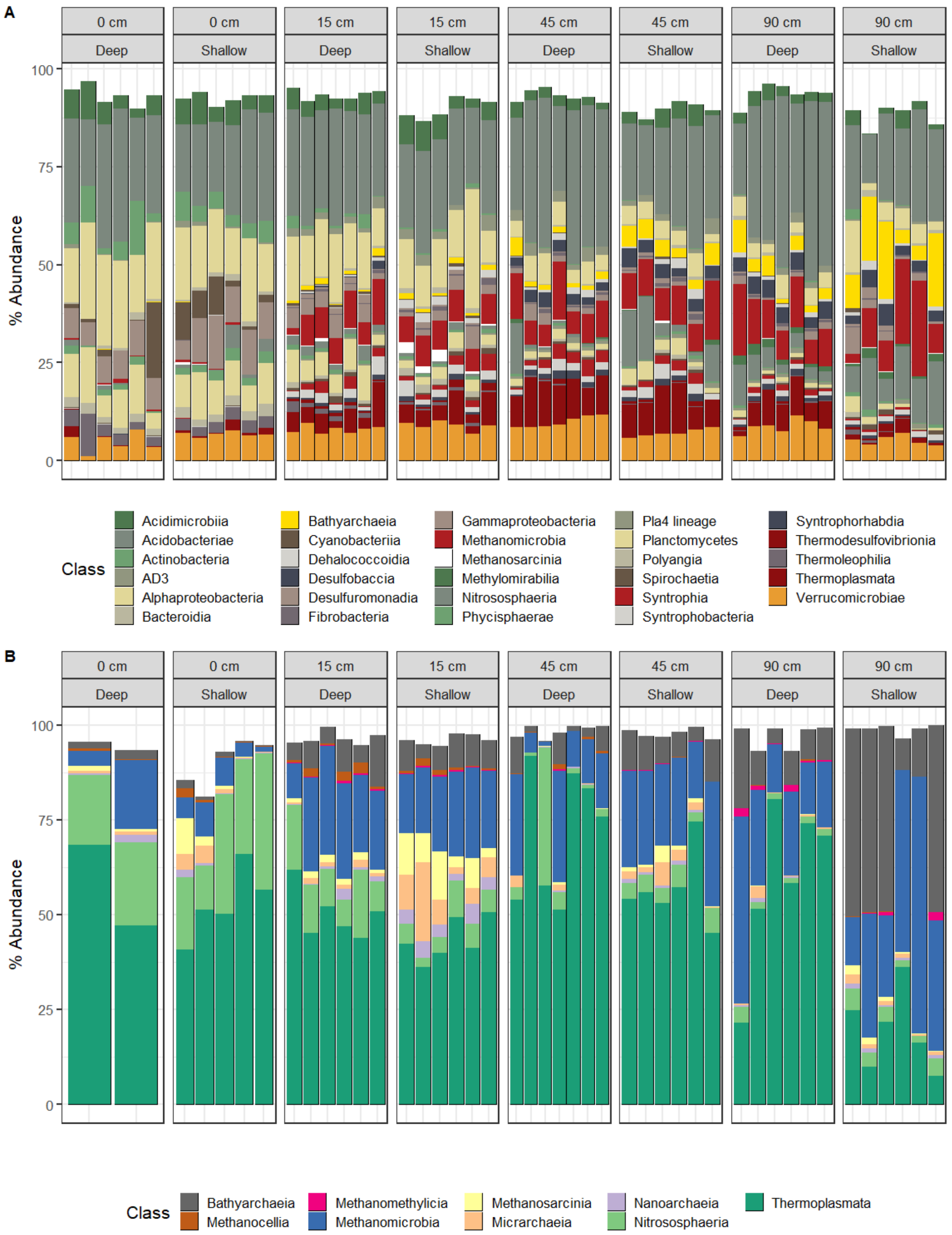

SI Figure 6: Relative abundances, at the class level, produced using **a.)** 16S universal amplicon data and **b.)** 16S archaeal-specific amplicon data. Abundances split by sampling and refusal depth. Taxa removed if the sum of their relative abundances (%) across all samples was <14.5.

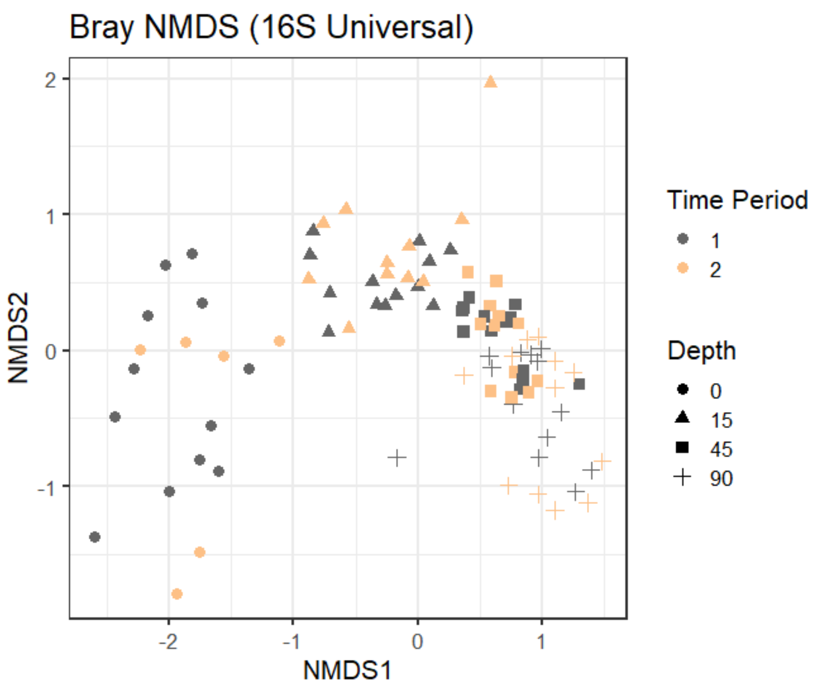

SI Figure 7: Ordination produced via non-metric multidimensional scaling using Bray-Curtis distance matrix calculated for 16S universal data. The main sampling expedition was conducted in August, 2020 (Time Period 1). A secondary sampling expedition, using the same study plots, was conducted a month later in September, 2020 (Time Period 2). Despite the fact that samples were collected a month apart, and not from the exact same point (anywhere within the 45 cm diameter plot), the community composition data between the two time points is not significantly different (adonis2, p>0.05).

SI Table 2: Thermocycling conditions and primers for both DNA and RNA-based gene expression and abundance assays.

Target	Primers	PCR Schedule	Ramp	Std. Curve
**mcrA**	*mcrA MLf*<br>GGTGGTGTMGGATTCACACART<br>AYGCWACAGC<br>*mcrA MLr*<br>TTCATTGCRTAGTTWGGRTAGTT	5 min at 95°C<br>30 sec at 95°C<br>30 sec at 52°C x40<br>45 sec at 72°C<br>5 min at 4°C<br>5 min at 90°C	2°C /s	R^2=0.98
**pmoA**	*pmoA 189f*<br>GGNGACTGGGACTTCTGG<br>*mb661r*<br>CCGGMGCAACGTCYTTACC	5 min at 95°C<br>30 sec at 95°C<br>35 sec at 52°C x40<br>50 sec at 72°C<br>5 min at 4°C<br>5 min at 90°C	1°C /s	R^2=0.98
**mmoX**	*mmoX 536f*<br>CGCTGTGGAAGGGCATGAAGCG<br>*mmoX 898r*<br>GCTCGACCTTGAACTTGGAGCC	5 min at 95°C<br>30 sec at 95°C<br>30 sec at 52°C x40<br>45 sec at 72°C<br>5 min at 4°C<br>5 min at 90°C	1.5°C /s	R^2=0.99

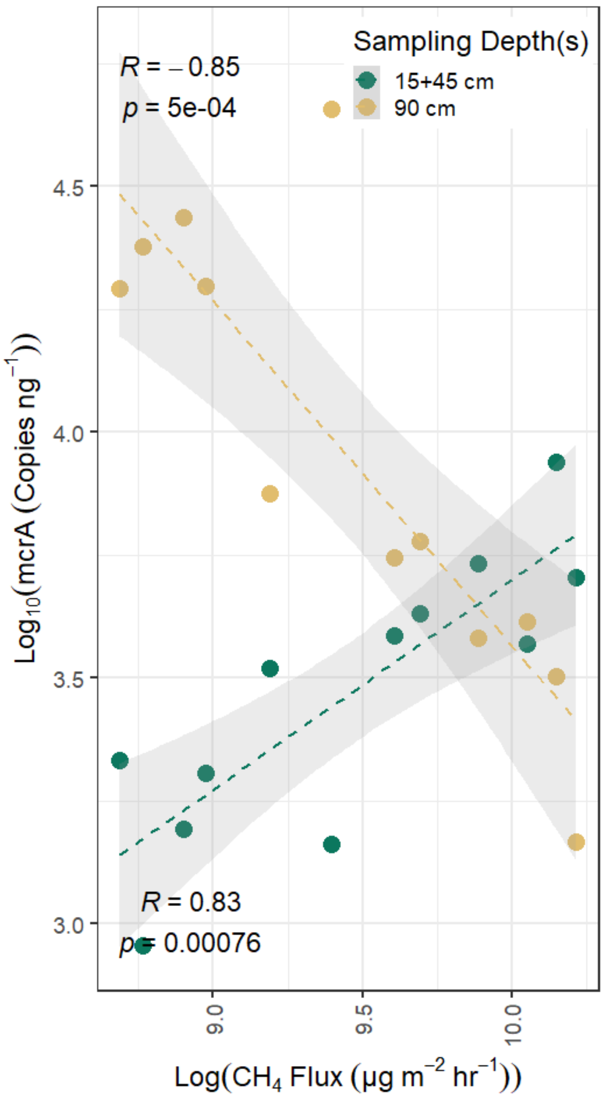

SI Figure 8: In shallow plots, methane flux was positively correlated (Pearson) with methanogen abundances (mcrA DNA) averaged at 15+45 cm and negatively correlated with abundances at 90 cm. In other words, high-fluxing shallow plots were characterized by high middle-depth (15+45 cm) methanogenic abundances and low abundances at 90 cm.
